# Equal Opportunity, Equal Work: Increasing Women's Participation in the U.S. President's Malaria Initiative Africa Indoor Residual Spraying Project

**DOI:** 10.9745/GHSP-D-17-00189

**Published:** 2017-12-28

**Authors:** Abigail Donner, Allison Belemvire, Ben Johns, Keith Mangam, Elana Fiekowsky, Jayleen Gunn, Mary Hayden, Kacey Ernst

**Affiliations:** a U.S. President's Malaria Initiative (PMI) Africa Indoor Residual Spraying (AIRS) Project, Abt Associates, Bethesda, MD, USA.; b U.S. President's Malaria Initiative (PMI), Washington, DC, USA.; c Results for Development, Washington, DC, USA.; d Jhpiego, Baltimore, MD, USA.; e Department of Epidemiology and Biostatistics, College of Public Health, University of Arizona, Tucson, AZ, USA.; f Research Applications Laboratory, National Center for Atmospheric Research, Boulder, CO, USA.

## Abstract

**Background:**

One of the primary control measures for malaria transmission is indoor residual spraying (IRS). Historically, few women have worked in IRS programs, despite the income-generating potential. Increasing women's roles in IRS requires understanding the barriers to women's participation and implementing measures to address them. The U.S. President's Malaria Initiative (PMI) Africa Indoor Residual Spraying (AIRS) Project is the largest implementer of IRS globally. To address gender inequity in IRS operations, PMI AIRS assessed the barriers to the participation of women and developed and implemented policies to address these barriers.

**Methods:**

The PMI AIRS Project initially identified barriers through a series of informal assessments with key stakeholders. PMI AIRS then implemented a series of gender-guided policies, starting in 2015, in Benin, Ethiopia, Ghana, Mali, Madagascar, Mozambique, Rwanda, Senegal, Zambia, and Zimbabwe. The policies included adapting physical work environments to ensure privacy for women; ensuring the safety of women in the workplace; guaranteeing safety and job security of women during pregnancy; and encouraging qualified women to apply for supervisory positions. The project collected routine programmatic data on staff, spray quality, and spray efficiency; data from 2012 through the end of 2015 were analyzed (up through 1 year after implementation of the gender policies). In addition, PMI AIRS conducted surveys in 2015, 2016, and 2017 before and after the spray campaigns in 4 countries to determine changes in gender norms among spray operators through questions about decision making and agency.

**Results:**

The PMI AIRS Project increased women's employment with the program. Specifically, women's employment increased overall from 23% in 2012 to 29% in 2015, with a 2015 range from 16% (Mali) to 40% (Madagascar). Growth among supervisor roles was even stronger, with the percentage of women in supervisory roles increasing from 17% in 2012 to 46% in 2015, with a 2015 range from 9% (Mali) to 50% (Madagascar). While the data showed that in most countries women sprayed fewer houses per day than men in 2015, the differences were not meaningful, ranging from 0.1 to 1.2 households per day. Gender norms shifted toward more egalitarian views in 2 of the 4 countries with survey data.

**Conclusion:**

Preliminary results suggest the PMI AIRS Project gender policies are increasing the engagement of women in all aspects of spray operations, especially in supervisory roles. Expansion of these policies to all countries implementing IRS and to malaria control implementation more broadly is recommended.

## Abstract (precis)

Promotion of gender policies led to increased hiring of women in supervisory roles in a large indoor residual spraying (IRS) program with no meaningful differences in IRS output between men and women spray operators.

## INTRODUCTION

The U.S. President's Malaria Initiative (PMI) Africa Indoor Residual Spraying (AIRS) Project implements indoor residual spraying (IRS), entomological monitoring, or both in 19 African countries. The PMI AIRS Project's goal is to reduce malaria morbidity and mortality through high-quality application of residual insecticides in the sleeping structures of malaria-endemic communities.^1^

In the past, women have had few operational roles in IRS programs. Yet the PMI AIRS country programs offer economic opportunities through paid positions that have the potential to enhance a woman's role in society. Engaging women in IRS may also increase communities' uptake of IRS, thus decreasing the need for revisits and increasing the efficiency of operations.

The PMI AIRS Project's IRS programs offer opportunities for paid employment to women in 19 African countries.

Several factors maximize the protective effect of IRS against malaria: susceptibility of mosquitoes to the insecticide, high coverage, and spray quality. High coverage of IRS relies on householders agreeing to have their homes sprayed. If too many individuals refuse, transmission can persist even where the mosquitoes are susceptible to the insecticide. Acceptance rates and factors related to acceptance of IRS vary by geographic region and include level of malaria transmission, sense of civic duty,^2^ perception of the effectiveness of IRS to reduce insects,^3^ distrust of political motivations,^4^ lack of understanding of IRS program objectives, and logistical constraints.^5^

In most African countries, including many sub-Saharan African countries, men traditionally are the authorities and have greater power to make household decisions,^6^^–^^8^ which may include whether or not to agree to have their home sprayed. However, spray activities occur during the daytime, when many men are out of the home for work. Women are often the ones at home when the IRS teams arrive to spray. It is therefore imperative that women understand and participate fully in the IRS process to ensure IRS programs' success and sustainability.

Although men are frequently seen as decision makers, gender norms also often support women's engagement in community building, health advocacy, and dissemination of knowledge through women's groups or women's roles as community health volunteers.^9^^,^^10^ Substantial information, education, and communication campaigns are conducted before any IRS activities; women are integral to these campaigns as community mobilizers (some are paid while others are volunteers, depending on the country). Thus, having women on IRS spray teams may garner even more support for the operations from women in the community. Moreover, employing women in these roles provides them the same opportunity as men for paid employment in a community program.

The PMI AIRS Project, with a mandate to promote gender equality and female empowerment at all levels of operations, developed a set of gender-based strategies in line with the U.S. Agency for International Development (USAID) Gender Equality and Female Empowerment policy.^11^ The intent was to (1) improve gender equality in employment by improving opportunities for women; (2) improve program outcomes; and (3) secure shared benefits of IRS, including economic benefits, across communities.

The PMI AIRS Project has developed a set of gender-focused employment strategies in line with USAID's Gender Equality and Female Empowerment policy.

This article describes how a large-scale malaria vector control project can mainstream gender equality. We use the definition of *gender equality* given by the Interagency Working Group on Gender^11^^,^^12^:

the state or condition that affords women and men equal enjoyment of human rights, socially valued goods, opportunities, and resources. Genuine equality means more than parity in numbers or laws on the books; it means expanded freedoms and improved overall quality of life for all people.

The expected results are improved employment opportunities for women and women's economic and social empowerment. Based on a literature review, the PMI AIRS Project further hypothesized that mainstreaming gender considerations into project operations would increase productivity and acceptance of IRS by more beneficiaries. Equality also was expected to spread the economic benefits of the project to a broader range of community members.

### Literature Review: Women in the Workforce

Although women have made great strides in education and employment globally, women are still less likely to hold leadership positions and more likely to live in poverty than men.^13^ To bring about long-term, sustained economic growth and healthy communities, governments and private-sector actors must engage both men and women. The recently adopted United Nations Sustainable Development Goals highlight this with Goal 5: Achieve gender equality and empower all women and girls.^14^ Entering the labor market and accessing formal employment is a route out of poverty for women and their families. According to the International Labour Organization, women account for 40% of the global workforce, yet their contribution receives little recognition^15^ and only half of all women are employed compared with 78% of men.^16^ Furthermore, women who work often work in sectors with lower pay, longer hours, and informal working agreements. This means that women make fewer monetary, social, and structural gains than men do. Moreover, nearly a quarter of women do unpaid family work.^8^

Entering the formal labor market is a route out of poverty for women and their families.

In African countries these gaps between women's and men's roles are even wider.^17^^–^^19^ Social norms still dictate that household chores and child-rearing be predominantly carried out by women.^15^ These norms limit women's economic opportunities and ability to participate in the workplace. A greater proportion of women who work in sub-Saharan Africa are limited to work in the informal sector.^7^ This situation leads to fewer investments in women's health: given men's greater earning power and control over income, women's health may not be valued as highly as men's health. The McKinsey Global Institute Report *The Power of Parity* confirms this. It found that although the level of gender equality in work in sub-Saharan Africa is comparable to that found in East and Southeast Asia, women in sub-Saharan Africa have the lowest levels of equality in access to essential services, financial services, and leadership roles. When women enter the formal employment sector, however, their contributions can lead to greater, systematic growth for their corporate employers. For example, in Chile, a study found that equalized gender distribution in the workplace increases hospitality, professionalism, worker efficiency, and motivation.^20^ Thus, promoting gender equality is an important aspect of business success and productivity improvement.^21^^–^^23^ Women bring rich and diverse perspectives to the workplace, including management styles which may differ from and be complementary to those of men.^24^

Although the scientific literature has called for a stronger emphasis on involving women in vector control for more than 30 years,^25^ these calls have until recently had little impact (Box). Historically, vector control programs have employed men because of the different roles that men and women traditionally fill in communities, with women being in charge of the domestic domain while men work outside the home.^26^

BOX **Research in Context**

**Evidence Available Before This Study**

The literature includes scarce evidence on the role of women in vector control. Publications that explicitly examine women's roles in vector control generally look at 2 issues: Some review the household, focusing on women as decision makers for uptake of protective measures for their families. Other publications look at women as volunteers in women's groups that provide community-level support through environmental management or distribution of larval control products. We identified no publications that examined attainment of gender equity in large-scale vector control programs. We searched PubMed, Scopus, and Web of Science in English with the search terms *women*, *vector or mosquito*, *indoor residual spray*, and *employment*.

**Implications of the Available Evidence**

There is little evidence that gender equity has been achieved within vector control programs. There is also scarce evidence of programs that have developed and evaluated policies focused on enhancing gender equity in vector control.

**Added Value of This Study**

The role of women in vector control activities, particularly paid vector control activities, is limited. Understanding mechanisms that work to improve women's participation is essential to reduce the gender gap in these activities. The PMI AIRS Project can serve as a model for implementing gender initiatives in vector control programs worldwide.

This affects not only women's employment in IRS operations but also the efficacy of IRS operations. That is because as the primary caretakers of the household, women are the first household members that vector control personnel such as IRS spray teams will encounter.^26^ However, cultural norms and safety precautions may not permit a woman to allow an unknown adult male to enter the house.^25^ This can impede vector control programs, which can miss houses or whole communities during program implementation. Including women in vector control programs may improve the acceptance and uptake of vector control strategies in some settings—thus increasing coverage and success.^26^

Including women in vector control programs may improve IRS acceptance and uptake in some settings.

Evidence suggests that when women are directly involved in all parts of health interventions within their community, acceptance and compliance, and thus impact, increase. Enhancing women's participation in development efforts such as water supply, sanitation, and agriculture has been integral to the success of some vector control programs.^27^^,^^28^ It is now essential to understand how women's participation in vector control programs affects program outcomes.

Research has shown that integrating women can not only improve business outcomes^20^^–^^24^ but also improve uptake and outcomes of malaria prevention projects.^29^^–^^32^ For example, in Thailand, a program that empowered a group of women to create malaria prevention plans and encouraged the use of long-lasting insecticide-treated bed nets resulted in significantly increased levels of malaria prevention behavior. Moreover, the women in the treatment villages felt more empowered to control malaria in addition to the empowerment that came from being a leader in the community and earning an income.^33^ These studies demonstrate the importance of considering gender and social norms when designing malaria prevention projects. However, most of these studies examine the role of women in their household-level decision-making abilities or how women engage as volunteers to motivate other community members to carry out malaria prevention strategies. The literature does not describe well engaging women in malaria prevention and control through the formal employment sector.

In this article, we outline the PMI AIRS Project's strategy to engage women in IRS operations. We present preliminary results of the strategy's impact on the percentage of women PMI AIRS employs and on changing gender norms.

## METHODS

### PMI AIRS Project Description and Gender Issues

The PMI AIRS Project has programs in 19 African countries: Angola, Benin, Burkina Faso, Burundi, Democratic Republic of the Congo, Ethiopia, Ghana, Kenya, Liberia, Madagascar, Malawi, Mali, Mozambique, Nigeria, Rwanda, Senegal, Tanzania, Zambia, and Zimbabwe. The first PMI AIRS Project contract spanned the years 2011 through 2014. During this time, PMI AIRS implemented spraying in 14 countries, spraying approximately 10 million houses and protecting more than 36 million people.

During these initial 3 years (2011–2014), PMI AIRS hired more than 88,000 seasonal workers. Only 25%, or 21,000, of these seasonal workers were women. In general, these women held lower-level and lower-paying positions such as washers or mobilizers. When PMI AIRS analyzed spray operations and operational sites, it found various reasons for the low number of women trained and hired. One was a strong social norm against women working on IRS activities, which have been perceived as “men's work.” The belief that women are not physically strong enough to do the work is a gender stereotype that discouraged PMI AIRS managers from offering women positions. Another barrier was project sites' lack of proper disposal facilities for menstruation supplies. In some cases, literacy and educational prerequisites prevented women from applying or qualifying for IRS-related work. But those requirements may not be necessary for all positions. For example, entomological data collection work is technical, yet has minimal education requirements. Lastly, some country programs require spray teams to camp overnight when spraying distant villages, and this is often not socially acceptable for women.

During its first 3 years, PMI AIRS hired 88,000 seasonal workers. Only 25% were women.

### PMI AIRS Project's Gender Policy Framework

**FIGURE 1.**
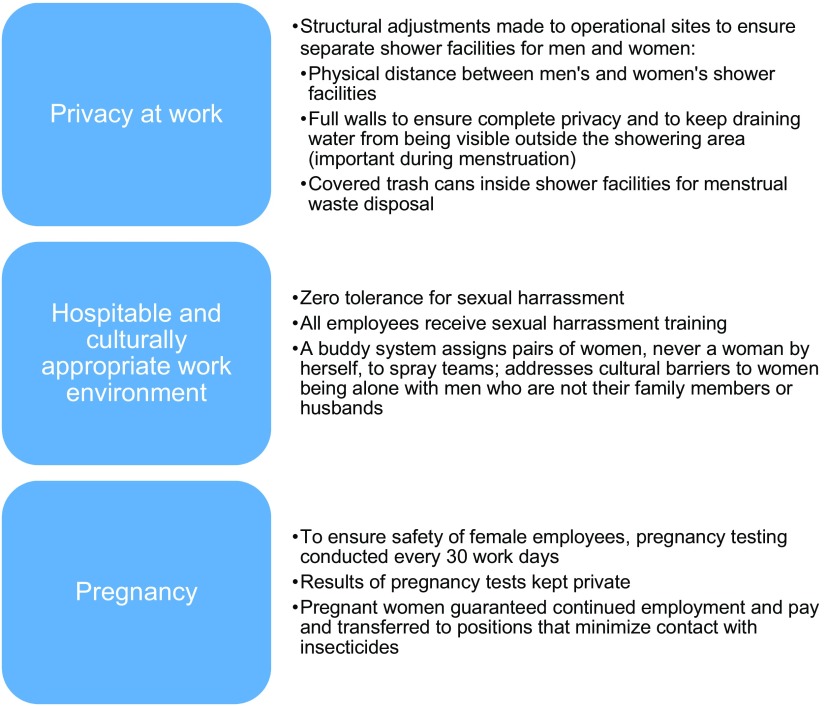
Overview of Gender-Focused Policies Implemented by the PMI AIRS Project

In 2014, PMI awarded Abt Associates another 3-year contract for PMI AIRS, to run from 2015 through 2018. The contract specified that the project must mainstream gender equality and female empowerment across project operations, in compliance with USAID's Gender Equality and Female Empowerment policy.^11^ During this contract, the PMI AIRS Project developed and implemented a multipronged policy to recruit and retain women in its program. Specifically, PMI AIRS implemented the strategies described below and summarized in Figure 1 starting in 2015 and will continue doing so through the end of the project in 2018.

In 2015, PMI AIRS developed and implemented strategies to recruit and retain women, including in supervisory positions.

#### Policy Focus 1: Privacy at the Work Environment

To attract and retain female employees, the PMI AIRS Project adapted its physical work environments to ensure privacy for women. IRS has been operating since the 1950s, and men traditionally design operational sites for men's needs. IRS operational sites need changing areas, bathrooms, and showers, because spray operators should change and shower after spraying to minimize the risk of insecticide contamination. In these sites, women had minimal privacy. They lacked separate changing areas because so few women used the facilities.

The PMI AIRS Project policy now requires that every operational site have separate changing areas, separate bathrooms with trash cans, and separate shower areas. Women's showers must be far from men's. Shower walls must start at the ground and be high enough to ensure privacy. Showers also must have proper drainage so that others cannot see the residual water; this is extremely important for women when they are menstruating. PMI AIRS has put these requirements into the environmental compliance checklists so all operational sites guarantee the same level of privacy. PMI AIRS spray campaigns cannot begin unless the environmental compliance officer verifies that operational sites are compliant.

PMI AIRS requires all operational sites to provide separate, private shower areas for men and women spray operators.

#### Policy Focus 2: Hospitable and Culturally Appropriate Work Environment

The PMI AIRS Project instituted policies to ensure a hospitable work environment that maximizes the safety of women in the workplace. PMI AIRS has zero tolerance for sexual harassment. All workers, both temporary and full-time staff, can anonymously report any sexual misconduct. At each operational site, PMI AIRS posts sexual harassment guidelines with a phone number to call to report any misconduct. The project has incorporated gender and sexual harassment awareness into training for government partners, supervisors, and seasonal employees.

PMI AIRS also instituted a buddy-system policy for female spray operators. Any woman on a team must have a second woman as a buddy on the team. The PMI AIRS Project put this policy in place to make women feel more comfortable, since spray days are extremely long and can involve long travel time to remote villages. Social norms in many countries where PMI AIRS operates discourage women from working all day alone with men who are neither their husbands nor family members.

PMI AIRS implemented a buddy system for female spray operators to make them more comfortable during long work or travel days.

#### Policy Focus 3: Safety and Job Security During Pregnancy

Exposure to insecticides is not safe for pregnant or lactating women, and spray teams spend hours in close proximity to insecticides. This is problematic for gender mainstreaming of IRS activities, because women of childbearing age are the women most likely to be attracted to and qualified for these positions. To uphold safety standards while attracting and retaining women on the team, the PMI AIRS Project implemented a pregnancy policy to protect women, their developing fetuses, and their breastfeeding infants.

All female seasonal workers take a pregnancy test every 30 days of work during the spray campaign. Procedures for testing and giving results ensure the woman's privacy. The project guarantees a position at the initial salary for any woman who has signed a contract and then becomes pregnant. The teams must find the pregnant woman another position without insecticide exposure, such as data verification assistant or mobilizer. Finally, although pregnancy before signing an employment contract is normally a disqualifier for women who aspire to work with PMI AIRS, the project makes an exception for women with prior spray campaign experience. The project employs these women in other positions for which they are qualified and that avoid exposure to insecticides. The objective is to retain and promote qualified women and enable professional growth and income generation during the childbearing years.

PMI AIRS guarantees employment at the initial salary for any woman who has signed an employment contract and then becomes pregnant.

#### Policy Focus 4: Women in Supervisory Positions

The PMI AIRS Project seeks to achieve gender equity. Gender equity is^6^:

the process of being fair to women and men, boys and girls. To ensure fairness, measures must be taken to compensate for cumulative economic, social, and political disadvantages that prevent women and men, boys and girls from operating on a level playing field.

The project identifies, mentors, and promotes talented women into supervisory positions. Project teams also implement affirmative action policies that give hiring priority to qualified female applicants for team leader and supervisor roles.

PMI AIRS identifies, mentors, and promotes talented women into supervisory positions.

All country teams set annual targets for and track and report on the percentage of supervisory roles held by women, with the eventual goal of gender parity among IRS supervisors. Women should have access to these positions and continue to be leaders in the community. Women who demonstrate that they can successfully take on these roles become role models to younger generations.

#### Policy Focus 5: Country-Level Flexibility

The PMI AIRS Project operates in a wide range of countries across sub-Saharan Africa, each with distinctive gender and social norms and IRS operations adapted to the local malaria epidemic and terrain. While all PMI AIRS country teams adhere to the basic policy standards outlined above, the project encourages country teams to include country-specific policies to advance gender equality and female empowerment within the project's vector control activities. A few examples of such policies follow:

**Ghana:** Ghana was the first country to mentor women for leadership roles and as part of an affirmative action plan. Because this policy worked so well and helped the Ghana team achieve its gender equality targets, PMI AIRS has made this a project-wide policy.

**Benin:** In Benin, the project team understood that to recruit more women, the team needed the support of local leaders who could create recruitment lists and make hiring decisions. The Benin team worked closely with local leaders to ensure that recruitment and hiring decisions were made in line with the project's gender equality goals.

**Rwanda:** The PMI AIRS Project in Rwanda currently hires the highest percentage of female spray operators of any PMI AIRS Project country. So the team decided to design a policy for hiring more women in other positions. The team has been working with the local authorities to change some job descriptions to be able to include both men and women. For example, to be a mobilizer, one currently must be a village chief. That excludes women. The team is changing the job description so that mobilizers can be village elders, which include men and women.

### Evaluation of PMI AIRS Project Gender Policy Implementation

To evaluate the impact of the project's gender policies, PMI AIRS examined several endpoints in Stata and Microsoft Excel using routine program data. This analysis included only countries that had at least 2 data points, to allow comparison before and after implementation of the gender policies.

During operations, teams routinely collect data for each spray operator on the number of houses sprayed each day, the number of refusals, and basic reasons for refusal. PMI AIRS also tracks team composition, permitting analysis by gender composition. The project uses standard forms to collect all data, which are ultimately aggregated and entered into a central database.

PMI AIRS collects routine data that can be analyzed by gender of spray operator.

#### Women's Employment in the PMI AIRS Project

We analyzed the data on the number of women receiving training for supervisory roles over 5 years in 9 of 10 project countries that adopted the project's gender policies. We excluded Zimbabwe because the PMI AIRS Project supports IRS implementation in Zimbabwe but does not manage the project or its hiring. Our analysis includes time before policy implementation (2012–2014) and for up to 1 year after the 2015 policy implementation (i.e., through 2016). The team calculated the percentage of women in positions such as team leader or site supervisor where they train and supervise others, to determine if the project trained and hired more women generally or hired women for higher-level positions. PMI AIRS conducted chi-square tests to determine whether the proportion of female supervisors rose between 2014 and 2015.

#### Women's Spraying Efficiency and Refusal Rates

Many people involved in IRS believe that female sprayers are not as efficient as men because of the hard labor involved. The project analyzed the average number of dwellings men and women sprayed in 9 countries in 2015. The analysis also explored the interaction between gender and IRS refusal. PMI AIRS used *t* tests to help determine if the average number of houses sprayed in each country varied by gender. Chi-square tests determined if refusal rates varied by gender.

#### IRS Team Members' Perception of Gender Norms

In 4 PMI AIRS Project countries—Ethiopia, Madagascar, Rwanda, and Zimbabwe—2,937 seasonal workers in randomly selected operational sites took a survey on gender norms and attitudes before the start and at the end of the 2015 spray campaign. The survey, adapted from the Gender Norm Attitudes Scale,^34^ examined spray operators' gender norms through questions about decision making and agency in the spray operators' households. The survey was translated into local languages and approved by the Abt Associates Internal Review Board (IRB) as well as by the appropriate local IRB in each country. Respondents indicated agreement or disagreement with the following statements:

1. Daughters should be sent to school only if they are not needed to help at home.
2. The only thing a woman can really rely on in her old age is her sons.
3. A good woman never questions her husband's opinions, even if she is not sure she agrees with them.
4. A woman must talk to her husband about her expenditures.
5. A woman should have her own money that she can use for what she would like to purchase.
6. If the woman works outside the home, her husband or partner does not need to help her with the daily housework.
7. A husband should not let his wife work outside the home, even if she would like to do it.
8. A woman must accept that her husband or partner beats her in order to keep the family together.
9. When it is a question of children's health, it is best to do whatever the father wants.
10. Women have the skills or the natural ability to make complex decisions.
11. It is ok for a supervisor to joke with his or her team members even if it makes them feel uncomfortable.

For these statements, less agreement with the statement that men have more rights and privileges than women reflects a more egalitarian perspective (coded as 1) compared with a more traditional perspective (coded as 0). PMI AIRS tallied the scores of individual items and then computed the mean of the scores expressed as a continuum from traditional beliefs (0) to egalitarian beliefs (1). Higher scores indicated more egalitarian beliefs. Country-level analyses examined changes in mean scores from preseason (before exposure to the PMI AIRS work environment and its gender policies) to postseason (after work with PMI AIRS). The project was not able to match pre- and postseason surveys for some respondents, however, due to errors in the assignment of anonymous identification numbers. Comparisons between scores used paired *t* tests for employees who could be matched as well as unpaired *t* tests which included all respondents. PMI AIRS stratified all analyses in Ethiopia, Madagascar, and Rwanda by demographic variables, including gender, head of household status, and educational status. Project staff in Zimbabwe did not collect this biographical data from respondents.

## RESULTS

The PMI AIRS Project continues to collect data on gender policy endpoints. Here we present the results of changes following the first year of implementation of the policies in 2015. We categorize the results and discussion below by changes in gender composition, spray quality, and social norms.

### Increasing Women's Employment in the PMI AIRS Project

**FIGURE 2.**
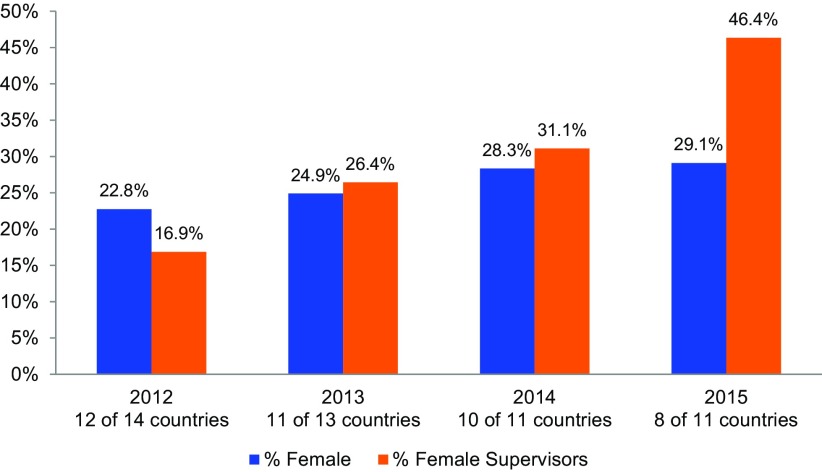
Percentage of Women Trained by the PMI AIRS Project, 2012 to 2015

**FIGURE 3.**
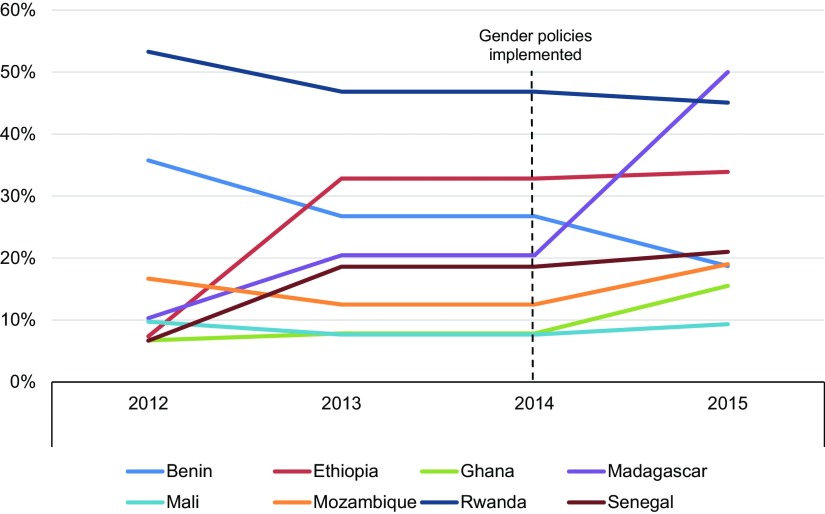
Percentage of Women in PMI AIRS Project Supervisory Positions by Country, 2012 to 2015

Table 1 shows the number of people PMI AIRS has trained since 2012, including the number of women trained and the percentage of people trained who were women. Figure 2 shows progress over time in recruitment of women and progress toward equity among those hired to implement IRS. The increases in the proportion of women participating across the time period, particularly in the category of supervisory positions, are noteworthy. Between 2012 and 2015, the overall proportion of women whom the PMI AIRS Project trained to deliver IRS increased by 6 percentage points, from 22.8% to about 29.1%. In that time, the number of women holding supervisory roles rose more than 29 percentage points: the percentage of women supervisors increased from 16.9% in 2012 to 31.1% in 2014, and then to 46.4% in 2015, after the gender policy was established. Figure 3 compares the increases in women supervisors from 2012 to 2015 across 8 PMI AIRS Project countries.

**TABLE 1.** PMI AIRS Project Training Data by Country and Year, 2012–2015

	2012	2013	2014	2015
**Benin**				
No. of people trained	1748	1543	2487	3333
No. of women trained	262	276	443	591
% trained who were women	15%	18%	18%	18%
**Ethiopia**				
No. of people trained	4213	3987	4390	4452
No. of women trained	992	1072	1472	1631
% trained who were women	24%	27%	34%	37%
**Ghana**				
No. of people trained	1265	1681	1657	1544
No. of women trained	200	233	300	292
% trained who were women	16%	14%	18%	19%
**Madagascar**				
No. of people trained	14,818	2241	3450	3302
No. of women trained	3583	482	1593	1337
% trained who were women	24%	22%	46%	40%
**Mali**				
No. of people trained	2371	2426	2066	1370
No. of women trained	303	409	271	226
% trained who were women	13%	17%	13%	16%
**Mozambique**				
No. of people trained	1953	1368	1677	2119
No. of women trained	596	303	625	624
% trained who were women	31%	22%	37%	29%
**Rwanda**				
No. of people trained	6062	9558	7801	8998
No. of women trained	1556	2738	2185	2581
% trained who were women	26%	29%	28%	29%
**Senegal**				
No. of people trained	1657	3973	1263	1287
No. of women trained	218	1221	218	397
% trained who were women	13%	31%	17%	31%
**Zambia**				
No. of people trained	–	–	1592	2105
No. of women trained	–	–	616	625
% trained who were women	–	–	39%	30%
**Total**				
No. of people trained	34,087	26,777	26,383	28,510
No. of women trained	7710	6431	7723	8304
% trained who were women	23%	25%	28%	29%

a No employee training data available for Zambia in 2012 and 2013, because until 2014, the PMI AIRS Project in Zambia provided only technical assistance.

### Evaluating Women's Spraying Efficiency and Refusal Rates

Analyses demonstrated that in most PMI AIRS countries, women sprayed fewer houses per day than men in 2016 (Table 2). In Benin, women sprayed the same average number of structures per day as men. Where there was an observed difference, the differences ranged from 0.1 household per day (Rwanda) to 1.2 households (Ghana) and were statistically significant (*P*<.05) only in Ghana, Ethiopia, Mozambique, and Zambia.

**TABLE 2.** Average Number of Structures Sprayed per Day by Country and Sex of Spray Operators, 2016

Country	Mean No. of Structures Sprayed per Day (N)		Difference Between Men and Women	*P* Value
	Men	Women		
Benin	14.6 (883)	14.6 (129)	0	.96
Ghana	17.1 (293)	15.9 (151)	1.2	<.001
Ethiopia	17.1 (1446)	16.3 (47)	0.8	<.001
Madagascar	14.3 (780)	14.1 (174)	0.2	.23
Mali	14.0 (695)	13.3 (299)	0.7	.15
Mozambique	8.0 (1089)	7.3 (403)	0.7	<.001
Rwanda	8.8 (534)	8.7 (729)	0.1	.42
Senegal	14.9 (340)	14.1 (157)	0.8	.49
Zambia	15.1 (741)	14.2 (374)	0.9	<.001

There were significant differences in refusal rates between men and women spray operators in 4 of the 9 countries for which 2016 data were available: Ghana, Mozambique, Senegal, and Zambia (Table 3). In Senegal, women spray operators had lower rates of refusals than men, while in the other 3 countries, women had higher rates of refusals. In Benin, Ethiopia, Madagascar, Mali, and Rwanda, refusal rates did not differ significantly between men and women.

**TABLE 3.** Average Spray Refusal Rates by Country and Sex of Spray Operators, 2016

Country	Refusal Rate^a^ (N)		Difference Between Men and Women	*P* Value
	Men Spray Operators	Women Spray Operators		
Benin	2.4% (883)	2.4% (129)	0.0%	.96
Ghana	1.0% (293)	1.4% (151)	−0.4%	<.001
Ethiopia	0.1% (1446)	0.1% (47)	0.0%	.60
Madagascar	0.3% (780)	0.2% (174)	0.1%	.06
Mali	0.7% (695)	0.7% (299)	0.0%	.47
Mozambique	11.3% (1089)	13.3% (403)	−2.0%	.005
Rwanda	0.1% (534)	0.1% (729)	0.0%	.71
Senegal^b^	0.5% (340)	0.4% (157)	0.1%	<.001
Zambia	1.2% (741)	1.3% (374)	−0.1%	<.001

a No. of households refusing to be sprayed per total structures found.

b Senegal's data were based on the number of rooms (not number of structures) that were *not* treated, due to refusals, over the number of rooms found.

### Changing IRS Team Members' Perception of Gender Norms

PMI AIRS identified 465 pairs of matching pre- and postseason surveys for Madagascar (38% of all preseason or baseline surveys), 628 pairs of surveys for Rwanda (79% of baseline surveys), 190 for Zimbabwe (79% of baseline surveys), and 115 for Ethiopia (17% of baseline surveys). Because of the high level of mismatch between surveys, we also examined the median survey scores of all respondents in each country and compared the pre- and postseason scores. As Table 4 shows, among all respondents, there was a 0.06 point increase in the mean gender index score in Madagascar (*P*=.48) and a 0.21 point increase in Rwanda (*P*=.03), where increases in scores indicate movement toward more egalitarian gender norms. There was a 0.37 point decrease in Zimbabwe (*P*=.04); and a 0.61 point decrease in Ethiopia (*P*=.001), where decreases in scores indicate movement toward more traditional gender norms.

**TABLE 4.** Changes in Gender Index Scores by Country, 2015

Country	No. Surveyed at Baseline	No. Surveyed at Endline	No. With Matched Baseline and Endline Surveys	% With Matched Baseline and Endline Surveys	Respondents Matched at Baseline and Endline				All Respondents	
					Gender Index Score^a^ at Baseline	Gender Index Score^a^ at Endline	Difference in Gender Index Score From Baseline^a^ (SE)	*P* Value	Adjusted Difference in Mean Gender Index Scores^b^ (SE)	*P* Value
Madagascar	1,227	937	465	38%	7.59	8.00	0.41 (0.11)	<.001	0.06 (0.08)	.48
Rwanda^c^	795	796	628	79%	7.17	7.47	0.30 (0.08)	<.001	0.21 (0.10)	.03
Zimbabwe	239	225	190	79%	8.37	8.04	−0.33 (0.16)	.048	−0.37 (0.18)^d^	.04
Ethiopia	676	499	115	17%	7.72	7.47	−0.25 (0.18)	.16	−0.61 (0.12)	< .001

Abbreviation: SE, standard error.

Notes: The PMI AIRS Project collected survey data among a sample of spray operators in 4 countries. Surveys were completed both before the 2015 spray season began (baseline or preseason) and after the season ended (endline or postseason).

a The gender index score is the average number of questions, out of a total of 11, answered in favor of gender egalitarianism.

b Adjusted results control for sex, previous experience working with the PMI AIRS Project (self-reported), ability to read, education, whether respondents live where they work, whether the respondents are heads of household, and (in Madagascar) district of the survey.

c Rwanda's 2015 spray season included 2 rounds of spraying.

d Covariates were not collected; results are not adjusted.

Surveys showed movement toward more egalitarian gender norms among PMI AIRS spray operators in some countries, and the opposite in other countries.

For the matched pairs, we also identified some significant differences in gender index scores after the spray campaign. We can see a statistically significant change toward more egalitarian gender norms in Madagascar, from a mean score of 7.59 preintervention to 8.00 postintervention (*P*<.001). In Rwanda also we find a statistically significant increase in the mean gender index score, from 7.17 to 7.47 (*P*<.001), suggesting a shift toward more egalitarian attitudes. In Zimbabwe, there was a statistically significant drop in the mean gender index score, meaning a shift toward more traditional gender norms, from 8.37 preintervention to 8.04 postintervention (*P*=.048). Ethiopia had no statistically significant change in the gender index score in the individuals with matched preseason and postseason surveys, but only a small percentage of respondents were able to be matched due to challenges in administering the survey in the field.

When we analyzed the survey data by gender of the spray operators, we found no statistically significant differences between men's and women's scores (Table 5). In Madagascar, women's preseason scores were higher (more egalitarian) than those of their male counterparts. The men's scores then increased more than women's did at the postseason survey, so that the men's scores approached the women's scores. In Ethiopia, we see men having an initially higher (more egalitarian) gender norms score, which dropped at postseason. Men's gender norms scores remained higher than women's in Ethiopia even with the men's decrease and the women's very slight increase. These can only be interpreted as associations.

**TABLE 5.** Changes in Gender Index Scores by Country and Sex of Spray Operators, 2015

Country	Sex	No. Surveyed at Baseline	No. Surveyed at Endline	No. (%) With Matched Baseline and Endline Surveys	Respondents Matched at Baseline and Endline				All Respondents	
					Gender Index Score^a^ at Baseline	Gender Index Score^a^ at Endline	Difference-in-Difference in Gender Index Scores (SE)	*P* Value	Adjusted Difference-in-Difference in Gender Index Scores^b^ (SE)	*P* Value
Madagascar	Men	686	617	147 (21)	7.32	7.78	0.16 (0.24)	.51	0.03 (0.17)	.88
	Women	537	320	319 (59)	8.19	8.49				
Rwanda^c^	Men	523	564	439 (84)	6.99	7.20	0.30 (0.17)	.09	0.14 (0.21)	.51
	Women	261	228	187 (72)	7.56	8.07				
Ethiopia	Men	532	390	106 (20)	7.80	7.53	0.29 (0.65)	.66	−0.15 (0.31)	.64
	Women	132	82	9 (7)	6.76	6.78				

Abbreviation: SE, standard error.

Notes: The PMI AIRS Project collected survey data among a sample of spray operators in 4 countries. Surveys were completed both before the spray season began (baseline or preseason) and after the season ended (endline or postseason). Zimbabwe is not included in this table because Zimbabwe's survey did not ask for the respondent's gender, due to government restrictions. Sex-disaggregated totals do not match overall total because demographic data were not collected from all respondents.

a The gender index score is the average number of questions answered, out of a total of 11, in favor of gender egalitarianism.

b Adjusted results control for sex, previous experience working with the PMI AIRS Project (self-reported), ability to read, education, whether respondents live where they work, whether respondents are heads of household, and (in Madagascar) the district of the survey.

c Rwanda's 2015 spray season included 2 rounds of spraying.

## DISCUSSION

Overall, there appear to be several promising indicators of success for the gender policies implemented by the PMI AIRS Project. As originally stated, we hypothesized that the gender-focused strategies would (1) improve employment opportunities for women, (2) increase productivity and acceptance of IRS by beneficiaries, and (3) spread the economic benefits of the project to a broader range of community members. Following implementation of the gender-focused policies, there was a substantive increase in the proportion of PMI AIRS supervisors who were women, indicating that access to employment opportunities had improved. However, there was variability by country. We found no substantive difference in acceptance of IRS by households in general, but in some cases a marginal increase in refusal for female spray operators. Finally, given the increased employment for women, we assume indirectly that the economic benefits were distributed to those who did not have such opportunities previously.

Though there were overall increases in the proportion of women employed in the PMI AIRS country programs between 2012 and 2015, there were some exceptions. In Ethiopia and Senegal, there was no significant increase in the proportion of women in supervisory roles between 2014 and 2015 after implementation of the gender policies. In addition, the overall proportion of women trained increased only slightly from 2014 to 2015. Although the increase in the percentage of women trained from 2012 to 2015 is only 6 percentage points, and there is less than a percentage point of increase since the PMI AIRS Project implemented the gender policies, the project has become more equitable in its hiring of women across all positions, with notable increases in the percentage of women in supervisory roles.

In terms of efficiency, our results indicate that perceptions that women are not able to carry out as much IRS work as men do not hold true across a range of settings in sub-Saharan Africa. We found marginal observed differences in the number of houses sprayed per day between men and women; these differences achieved statistical significance in 4 of the 9 countries where data were available. PMI AIRS, however, considers these observed differences to be too small to have an impact on programmatic efficiency. Meanwhile, contrary to our hypothesis that women would receive a lower rate of refusal than men, the opposite was true in several countries evaluated. On average, men experienced a higher rate of refusals than women in Senegal, while women experienced a higher rate of refusals in Ghana, Mozambique, and Zambia. While these differences are statistically significant, they are again quite small and unlikely to have a meaningful impact on operational results.

Differences found in spray efficiency between men and women were considered too small to affect overall efficiency of the program.

Discussions with field personnel indicate several possible explanations for the higher refusal rates observed when women act as spray operators, which are consistent with previous literature. It has been documented that women tend to be less assertive than men.^35^^,^^36^ While women may be more likely to accept other women into their home for spraying, female sprayers may be less assertive when approaching households for spraying. In male-dominated societies, it may be less acceptable for a woman to refuse a male request than a request presented by a female. The level of comfort that a female householder may have with a female spray operator may lead her to decline spraying more readily. Culturally, menstruation is considered taboo in many countries, and in some cases, women are shunned while menstruating.^37^^,^^38^ In some areas, women spray operators may be turned away by male householders because social norms deem menstruation “unclean” and men are reluctant to have an unknown woman in the home who may be menstruating. PMI AIRS will investigate this gap in refusal further to determine key factors that can be addressed. Training spray operators on recruitment strategies is a potential solution that could improve not only women's but also men's ability to obtain consent to spray a home.

Gender norms pre- and postimplementation of the gender-focused policies shifted toward more egalitarian views in 2 of 4 countries under study. Employees in Zimbabwe showed a significant shift toward more traditional gender norms. PMI AIRS will repeat this analysis to better understand the link between seasonal employment and stated expression of egalitarian or traditional gender norms. One limitation of the current gender norms survey is that it is focused broadly on changes in core gender norms that are not related to vector control. It is likely that gender mainstreaming in vector control may alter perceptions about women in vector control more readily than it affects broad-based gender norms. Therefore, in future surveys, additional items will be included to obtain information on perceptions surrounding women's role in vector control activities.

Since this is a multiyear assessment, the project will continue to analyze the data, but the preliminary results suggest that the gender-focused strategies are increasing the engagement of women in all aspects of the spray operations, in particular in supervisory roles. Moderate shifts in reported gender norms among seasonal employees participating in the gender norms survey are also encouraging. The project recognizes that it employs seasonal workers for very short periods of time. Spray campaigns last approximately 35 days out of the year. Therefore, we did not expect to see a large change in gender norms after the first year of the project's gender policies. Further work is needed to investigate the reasons for higher IRS refusal rates for women-led teams.

## CONCLUSION

The PMI AIRS Project has demonstrated that it is possible to integrate gender considerations into project operations. The project has shown that by designing IRS operations in a manner that recognizes that women can play key roles, IRS campaigns can become gender equitable, attract and hire more women, and give women an opportunity to gain professional skills. Yet there is still room to improve the project's responsiveness to women's needs.

Gender-focused policies in IRS programs can increase opportunities for women to gain formal employment, income, and professional skills.

It has been harder to quantify the effect of increasing gender equality on spray operations. The project has collected data that indicate increased hiring of women in all positions, but the data that measure whether increased participation by women in spray operations changes either spray quality or social norms is not conclusive. Since this is a new initiative, the PMI AIRS Project will continue to monitor and quantify these questions.

## Notes

**Peer Reviewed**

**Funding:** This work was conducted by the United States Agency for International Development's Africa Indoor Residual Spraying Project, with financial support from the U.S. President's Malaria Initiative.
